# Native Cellulose: Structure, Characterization and Thermal Properties

**DOI:** 10.3390/ma7096105

**Published:** 2014-08-25

**Authors:** Matheus Poletto, Heitor L. Ornaghi Júnior, Ademir J. Zattera

**Affiliations:** Laboratory of Polymers (LPOL), University of Caxias do Sul (UCS), Francisco Getúlio Vargas 1130, Caxias do Sul, Rio Grande do Sul 95070-560, Brazil; E-Mails: ornaghijr.heitor@gmail.com (H.L.O.J.); ajzatter@ucs.br (A.J.Z.)

**Keywords:** natural fibers, cellulose, crystallinity, XRD, FTIR, thermal stability

## Abstract

In this work, the relationship between cellulose crystallinity, the influence of extractive content on lignocellulosic fiber degradation, the correlation between chemical composition and the physical properties of ten types of natural fibers were investigated by FTIR spectroscopy, X-ray diffraction and thermogravimetry techniques. The results showed that higher extractive contents associated with lower crystallinity and lower cellulose crystallite size can accelerate the degradation process and reduce the thermal stability of the lignocellulosic fibers studied. On the other hand, the thermal decomposition of natural fibers is shifted to higher temperatures with increasing the cellulose crystallinity and crystallite size. These results indicated that the cellulose crystallite size affects the thermal degradation temperature of natural fibers. This study showed that through the methods used, previous information about the structure and properties of lignocellulosic fibers can be obtained before use in composite formulations.

## 1. Introduction

The last few decades have revealed the growing interest of industry and scientists on the research and development of polymeric and composite materials from renewable sources [1,2,3,4]. As the most abundant component in most plants, cellulose is an almost inexhaustible polymeric raw material from renewable sources. Natural cellulose-based materials, such as wood and natural fibers, have been used as engineering materials for thousands of years, and their use currently continues, as demonstrated by the huge number of forest product-based worldwide industries. However, what makes cellulose such an important material for the development polymeric and composite materials? The cellulose macromolecule is made up of repeating glucose units that generate surprising specificity and impressively diverse architectures, reactivities and functions [5]. The reactions and properties of native cellulose are determined by the isolation process used, the number of inter- and intra-molecular hydrogen bonds, the chain lengths, the chain length distribution, the crystallinity and the distribution of functional groups within the repeating units and along the polymer chains [5,6,7]. These important parameters make cellulose a unique material. Therefore, knowledge of and understanding the properties of native cellulose before their use in composites can result in composites with better mechanical and thermal properties.

In order to better understand the relationships between native cellulose structure and properties, the aim of this work was to establish the main structural differences between six vegetal fibers (curaua, ramie, kenaf, jute, sisal and buriti) and four wood fibers (*Pinus elliottii*, *Eucalyptus grandis*, *Mezilaurus itauba* and *Dipteryx odorata*) commonly used as reinforced fillers in composite materials through X-ray diffraction (XRD) analysis, Fourier transform infrared (FTIR) spectroscopy and thermogravimetric analysis (TGA).

## 2. Experimental Section

### 2.1. Materials

The wood flour samples used in this study were obtained from wastes of the lumber industry in Brazil. The species investigated were *Pinus elliottii* (PIE), *Eucalyptus grandis* (EUG), *Mezilaurus itauba* (ITA) and *Dipteryx odorata* (DIP). The others six vegetal fibers also used in this study were: *Hibiscus cannabinus* (kenaf), *Corchorus capsularis* (jute), *Agave sisalana* (sisal), *Ananas erectifolius* (curaua), *Boehmaria nivea* (ramie) and *Mauritia flexuosa* (buriti). Kenaf, jute and sisal fibers were supplied by Tapetes São Carlos Technology (from São Carlos, SP, Brazil) as sheets. Curaua fiber was obtained from Centro de Apoio a Projetos de Ação Comunitária (CEAPAC), a support center for community action projects in Santarem/PA, Brazil. Ramie roving was purchased from Sisalsul Fibras Naturais (São Paulo, SP, Brazil), and buriti fiber was obtained from Sisalsul Fibras Naturais (Caxias do Sul, RS, Brazil). All of the fiber samples were dried at 105 °C for 24 h in a vacuum oven before the tests. The samples were grounded in a knife mill, and the average fiber particle length used in all analyses was around 200 μm.

### 2.2. Fourier Transform Infrared (FTIR) Spectroscopy

FTIR spectra were obtained by means of a Nicolet IS10spectrometer (Thermo Scientific, Waltham, MA, USA). Natural fiber powder samples of each species (5 mg) were dispersed in a matrix of KBr (100 mg), followed by compression to form pellets. The sample collection was obtained using 32 scans, in the range of 4000 to 400 cm^−1^, at a resolution of 4 cm^−1^. Three different measurements for each fiber were evaluated, and the average value was considered.

### 2.3. X-ray Diffraction

X-ray diffractograms were collected using a sample holder mounted on a XRD-6000 diffractometer (Shimadzu, Kyoto, Japan) with monochromatic CuKα radiation (λ = 0.15418 nm). The generator was utilized at 40 kV and 30 mA, and the intensities were measured in the range of 5° < 2θ < 30°, typically with scan steps of 0.05° at 2 s/step (1.5°/min). Peak separations were carried out using Gaussian deconvolution. After deconvolution, it is possible to calculate and compare several parameters. The *d*-spacings were calculated using the Bragg equation. In this study, the *Z*-discriminant function developed by Wada and Okano [8] for the determination of the crystalline structure (monoclinic and triclinic) of cellulose in natural fibers was used. By employing discriminant analysis, it is possible to categorize cellulose as Type I_α_ or I_β_ [8]. The *Z*-value indicates whether cellulose is I_α_ or I_β_. The function that discriminates between I_α_ or I_β_ is given by Equation (1):

*Z* = 1693*d*_1_ – 902*d*_2_ − 594 (1)

where *d*_1_ is the d-spacing of the peak (1–10); *d*_2_ is the d-spacing of the peak (110); and *Z* > 0 indicates I_α_; while *Z* < 0 indicates the I_β_ dominant type [8].

The crystalline index proposed by Hermans *et al*. [8,9] is presented in Equation (2):

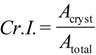
(2)
where *Cr.I.* is the crystalline index; *A*_cryst_ is the sum of crystalline band areas; and *A*_total_ is the total area under the diffractograms.

The second approach used to determine the crystalline index, Equation (3), was the empirical method proposed by Segal *et al*. in 1959 [8,10], which is:

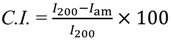
(3)
where *I*_200_ is the maximum intensity of the (200) lattice diffraction; and *I*_am_ is the intensity diffraction of the amorphous band.

The apparent crystallite size (*L*), shown in Equation (4), was calculated using the Scherrer equation [9]:

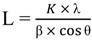
(4)
where *K* is a constant of value 0.94; λ is the X-ray wavelength (0.1542 nm); β is the half-height width of the diffraction band; and θ is the Bragg angle corresponding to the (200) plane.

### 2.4. Thermogravimetric Analysis

The thermogravimetric analysis was carried out on a TGA50 analyzer (Shimadzu, Kyoto, Japan) under constant nitrogen flow (50 mL/min), from 25 to 800 °C, at a heating rate of 10 °C/min. Approximately 10 mg of each sample were used.

## 3. Results and Discussion

### 3.1. Fibers Chemical Composition

Table 1 shows the composition of the fibers used in this study based on the literature [11,12,13,14]. Wood fibers present higher fractions of lignin and holocellulose (cellulose and hemicellulose) than the others natural fibers. Extractive content in wood is also higher than natural fibers. Tropical woods, such as *Dipteryx odorata* and *Mezilaurus itauba*, showed extractive contents three-times higher than temperate woods, such as *Eucalyptus grandis* and *Pinus elliottii*. On the other hand, vegetal fibers showed higher cellulose content associated with lower fractions of lignin and extractives.

**Table 1:** Chemical composition of the fibers used in this study obtained from the literature [11,12,13,14].

Fibers	Cellulose (wt%)	Hemicellulose (wt%)	Lignin (wt%)	Pectin (wt%)	Waxes (wt%)	Extractives (wt%)
	Holocellulose (wt%)					
*Eucalyptus grandis*	61.3–64		31–33	–	–	3.9–4.3
*Pinus elliottii*	60–62.3		32.8–35	–	–	4.4–4.6
*Dipteryx odorata*	56.5–57.7		30–31	–	–	11–11.2
*Mezilaurus itauba*	56.8–58.8		27.7–28	–	–	13–14.3
Curaua	71–74	9.9–21	7.5–11	–	0.79–0.9	2.5–2.8
Jute	45–71	13.6–21	12–26	0.2–10	0.5	2
Kenaf	31–72	20.3–23	9–19	3–5	–	2–5
Ramie	68.6–91	5–16.7	0.6–0.7	1.9–2	0.3	6
Sisal	65–67	12	9.9	2–10	0.3–2	0.8–2
Buriti	65–71		21–27	–	–	5.4–6.0

The mechanical and physical properties of the fibers are mainly influenced by their composition, mainly cellulose, hemicellulose and lignin [15,16]. As an example, higher tensile strength and higher thermal stability are obtained for fibers that contain more crystalline cellulose [10]. Hemicellulose is one of the fiber components responsible for the initial thermal degradation behavior and is also associated with the moisture content [15]. Therefore, fibers containing high hemicellulose content should absorb more moisture and degrade at a lower temperature. In addition, higher quantities of extractives may promote fiber degradation at low temperatures [16]. Thus, the degradation characteristics of natural fibers may be estimated based on their chemical composition.

### 3.2. FTIR Spectroscopy

**Figure 1:**
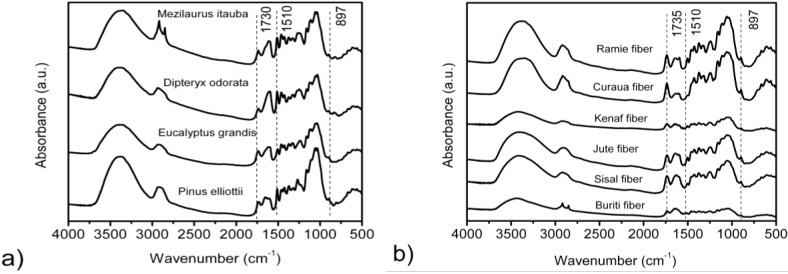
FTIR spectra of the wood fibers (**a**); and vegetal fibers (**b**) studied.

The FTIR spectra for wood fibers and vegetal fibers studied are shown in Figure 1a,b, respectively. It can be observed that there is a strong broad band at around 3400 cm^−1^, which is assigned to different O–H stretching modes, and another two bands at around 2920 and 2850 cm^−1^, related to asymmetric and symmetric methyl and methylene stretching groups present in the spectra of all of the fiber components, but most notably in the spectra for cellulose [16,17]. However, these two bands are more prominent in the ITA spectra at 2916 and 2852 cm^−1^, respectively, and buriti fiber spectra at 2918 and 2849 cm^−1^, respectively. This might be attributed to the higher extractive contents in these two fibers, as can be seen in Table 1, since some compounds in organic extractives, such as fatty acid methyl esters and phenolic acid methyl esters, contain methyl and methylene groups [18,19,20]. In the fingerprint region, the bands at 1595, 1510 and 1270 cm^−1^ are assigned to C=C, C–O stretching or bending vibrations of different groups present in lignin [16,17,18,19,20]. The bands at 1460, 1425, 1335, 1220 and 1110 cm^−1^ are characteristic of C–H, C–O deformation, bending or stretching vibrations of many groups in lignin and carbohydrates [18,19,20]. The bands at 1735, 1375, 1240, 1165, 1060 and 1030 cm^−1^ are assigned to C=O, C–H, C–O–C and C–O deformation or stretching vibrations of different groups in carbohydrates [16,17,18].

The hydrogen bonds are considered to be responsible for various properties of native cellulose, lignin and, of course, natural fiber itself [7,9]. Thus, the closer the cellulose chains, the greater the interaction between the adjacent chains, resulting in more and stronger hydrogen bonds, which can lead to greater packing of cellulose chains, resulting normally in fibers with higher mechanical and thermal properties [16,17].

The intramolecular hydrogen bond in a phenolic group in lignin is observed at around 3568–3577 cm^−1^ [21]. In cellulose, an intramolecular hydrogen bond vibration appears at around 3432 cm^−1^ [7,22]. Another intramolecular hydrogen bond in cellulose normally occurs at 3342 cm^−1^ [22]. The two characteristic bands assigned to the two crystalline cellulose allomorphs, cellulose I_α_ and cellulose I_β_, also occur in the region of 3220–3280 cm^−1^ [9]. A very small peak, normally shifted to lower wavenumbers, at 3221 cm^−1^, was attributed to hydrogen bonds only in cellulose I_α_ [7,9]. The band at 3221 cm^−1^ is assigned to the intramolecular hydrogen bonds present only in triclinic I_α_ cellulose, whereas the band at close to 3277 cm^−1^ is proportional to the amount of monoclinic cellulose I_β_. The energy of the hydrogen bonds *E*_H_ for several OH stretching bands has been calculated using Equation (5) [23]:

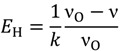
(5)
where ν_O_ is the standard frequency corresponding to free OH groups (3650 cm^−1^); ν is the frequency of the bonded OH groups; and *k* is a constant (1/*k* = 2.625 × 10^2^ kJ).

The hydrogen bond distances *R* are obtained using Equation (6) proposed by Pimentel and Sederholm as follows [24]:

*∆*ν (cm^−1^) = 4430 × (2.84 − *R*) (6)

where *∆*ν *=* ν_O_ – ν; ν_O_ is the monomeric OH stretching frequency, which is taken to be 3600 cm^−1^; and ν is the stretching frequency observed in the infrared spectrum of the sample.

The energy of hydrogen bonds and hydrogen bond distances for all of the fibers studied are presented in Table 2. Curaua, jute and kenaf fibers present a lower energy of hydrogen bonds values at 3567 cm^−1^. This could be associated with a higher quantity of absorbed water in the structure of these fibers, since the band at 3567 cm^−1^ is also assigned to the weakly absorbed water [22,25]. On the other hand, *Pinus elliottii*, *Dipteryx odorata* and *Mezilaurus itauba* showed the higher energy of hydrogen bonds values at 3567 cm^−1^. According to Table 1, these wood fibers had higher quantities of lignin, which may contribute to forming several intramolecular hydrogen bonds between neighbor phenolic groups in lignin, reducing the distances between the neighbor phenolic groups, as can be seen by the lower hydrogen bond distance values for these three fibers in comparison with the other fibers at 3567 cm^−1^. The energy of hydrogen bonds values for bands at 3423 and 3342 cm^−1^ are similar for all fibers studied. However, considering these two bands from woods, *Dipteryx odorata* showed higher energy values, while buriti presents higher values for the vegetal fibers, which may indicate a higher number of intramolecular hydrogen bonds in cellulose in these two fibers. These higher values were associated with lower hydrogen bond distances, which may contribute to higher interactions between intramolecular cellulose chains. The energy values at 3278 and 3221 cm^−1^ related to the cellulose allomorphs forms are similar for all fibers studied.

**Table 2:** Energy of the hydrogen bonds and hydrogen bond distance for the fibers studied.

Fibers	3567 cm^−1^		3423 cm^−1^		3342 cm^−1^		3278 cm^−1^		3221 cm^−1^	
	E_H_ (kJ)	R (Å)	E_H_ (kJ)	R (Å)	E_H_ (kJ)	R (Å)	E_H_ (kJ)	R (Å)	E_H_ (kJ)	R (Å)
*Eucalyptus grandis*	6.185	2.832	16.182	2.800	22.438	2.782	26.574	2.768	30.349	2.756
*Pinus elliottii*	6.329	2.831	16.757	2.799	22.007	2.782	26.394	2.768	30.314	2.757
*Dipteryx odorata*	6.401	2.831	16.613	2.799	22.438	2.781	26.610	2.768	30.874	2.754
*Mezilaurus itauba*	6.473	2.831	16.325	2.800	22.295	2.781	26.753	2.767	30.493	2.756
Curaua	5.969	2.833	16.038	2.801	21.935	2.782	26.746	2.767	30.997	2.754
Jute	5.980	2.833	16.038	2.801	21.827	2.783	26.538	2.768	30.666	2.755
Kenaf	5.667	2.833	16.253	2.800	22.043	2.782	27.041	2.766	30.781	2.755
Ramie	6.048	2.832	16.325	2.800	22.100	2.782	26.782	2.767	31.140	2.754
Sisal	6.156	2.832	16.253	2.800	21.863	2.783	26.538	2.768	31.148	2.754
Buriti	6.185	2.832	16.253	2.800	22.366	2.781	27.041	2.766	31.184	2.753

The band at around 1420–1430 cm^−1^ is associated with the amount of the crystalline structure of the cellulose, while the band at 898 cm^−1^ is assigned to the amorphous region in cellulose [6]. The ratio between the two bands was defined as an empirical crystallinity index proposed by Nelson and O’Connor [26] as a lateral order index (LOI). The ratio between the bands at 1372 and 2900 cm^−1^, also proposed by Nelson and O’Connor (1964) [26] to be the total crystalline index (TCI), was used to evaluate the infrared crystallinity (IR) ratio. Considering the chain mobility and bond distance, the hydrogen bond intensity (HBI) of cellulose is closely related to the crystal system and the degree of intermolecular regularity, that is, crystallinity, as well as the amount of bound water [27]. The ratio between the absorbance bands at 3400 and 1320 cm^−1^ was used to study the HBI of the fibers. The results obtained are presented in Table 3.

**Table 3:** Infrared crystallinity ratio and hydrogen bond intensity of the fibers studied. HBI, hydrogen bond intensity.

Fibers	IR crystallinity ratio		HBI
	H1372/H2900 (TCI)	H1429/H897 (LOI)	A3400/A1320
*Eucalyptus grandis*	0.608 ± 0.01	3.172 ± 0.02	1.440
*Pinus elliottii*	0.474 ± 0.01	2.299 ± 0.04	1.598
*Dipteryx odorata*	0.389 ± 0.02	3.137 ± 0.03	1.508
*Mezilaurus itauba*	0.237 ± 0.03	2.060 ± 0.01	1.523
Curaua	1.300 ± 0.01	1.070 ± 0.01	1.132
Jute	1.150 ± 0.03	0.990 ± 0.01	1.207
Kenaf	1.190 ± 0.01	0.930 ± 0.02	1.119
Ramie	1.240 ± 0.01	1.050 ± 0.01	1.426
Sisal	1.150 ± 0.02	0.970 ± 0.03	1.625
Buriti	1.150 ± 0.01	0.780 ± 0.05	2.241

The TCI is proportional to the crystallinity degree of cellulose [28], and the LOI is correlated to the overall degree of order in the cellulose [28,29]. EUG showed the highest TCI and LOI values, indicating the highest degree of crystallinity and a more ordered cellulose structure than the other species. On the other hand, ITA presented the lowest TCI and LOI values, which may indicate that the cellulose of this wood is composed of more amorphous domains when compared with the other three species evaluated, while PIE and DIP presented intermediate values. Another interesting finding is that DIP presented a high LOI value, similar to that of EUG, which is associated with a laterally ordered cellulose structure. However, EUG had the lowest HBI value. The PIE sample had the highest HBI value, while DIP and ITA presented similar values.

For vegetal fibers, curaua showed the highest TCI value, followed by ramie and kenaf, while jute, sisal and buriti had similar values. It is possible that curaua showed higher cellulose crystallinity when compared with the others fibers, and jute, sisal and buriti may present more amorphous domains in the cellulose structure. When the values of LOI were compared, curaua also showed the highest cellulose crystallinity, followed by ramie fiber. Jute, kenaf and sisal showed similar values, while buriti presents the lowest value. Buriti presents an HBI value almost two-times higher than the others vegetal fibers. This result seems to indicate that buriti has a higher cellulose crystallinity than the other fibers; however, the ratio between the bands at 3400 and 1320 cm^−1^ also represent the amount of bound water in the fiber structure, and based on the TCI and LOI results, it might be inferred that the amount of bound water in buriti may be influenced in this result. Therefore, great care needs to be taken when using ratios between FTIR bands to compare cellulose crystallinity and others parameters using FTIR spectroscopy. The influence of others fiber components, such as extractives, hemicellulose and lignin associated with higher quantities of bound water, may contribute to masking the results. As an example, Ornaghi, Jr. *et al*. [15] observed that the band at 2900 cm^−1^ may be also associated with linear chain extractives derived from hydrocarbonates, and fibers with higher extractive content can show higher values in this specific band, decreasing the calculated total crystallinity value.

### 3.3. XRD Results

**Figure 2:**
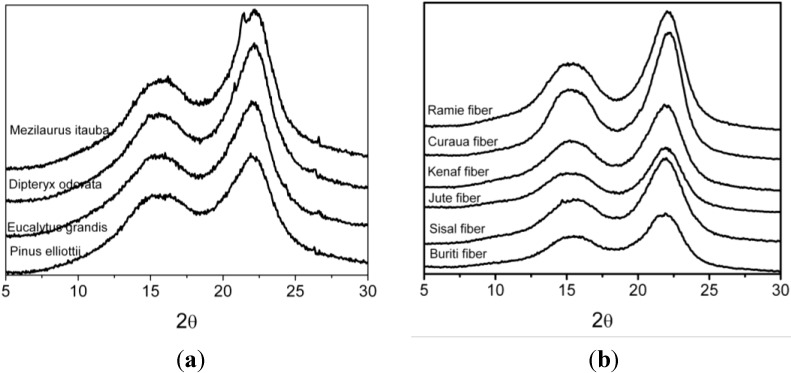
X-ray diffractograms of (**a**) wood species and (**b**).vegetal fibers studied

The X-ray diffractograms of the wood and natural fibers studied are shown in Figure 2a,b, respectively. In order to examine the intensities of the diffraction bands, to establish the crystalline and amorphous areas more exactly and to determine the crystallite size, the diffractograms were deconvoluted using Gaussian profiles. The peak intensities and peak broadening differ from one species to another. The more pronounced difference occurs at the peak range between 21.90° and 22.20° 2θ reflection assigned with a crystallographic plane of cellulose. Crystallographic planes are labeled according to the native cellulose structure, as described by Wada *et al*. [8]. After deconvolution, all diffractograms show the 14.5°–15.3° 2θ reflection assigned to the (1–10) crystallographic plane, the 15.7°–16.30° 2θ reflection assigned to the (110) crystallographic plane, the 18.30°–18.40° 2θ reflection assigned to the amorphous phase and the 21.90°–22.20° 2θ reflection assigned to the (200) crystallographic plane of cellulose I [8,9].

**Figure 3:**
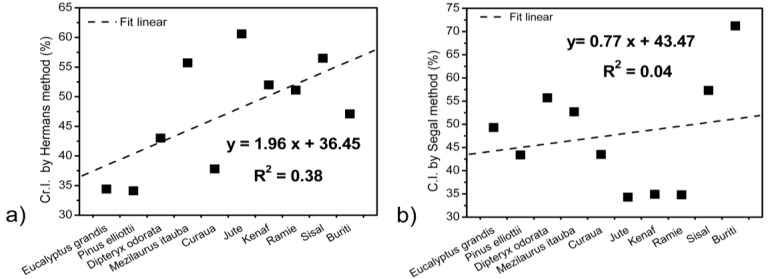
Liner regression comparing the Hermans (**a**); and Segal (**b**) methods.

The degree of cellulose crystallinity is one of the most important crystalline structure parameters. The rigidity of cellulose fibers increases and their flexibility decreases with increasing the ratio of crystalline to amorphous regions [10]. The crystallinity index calculated according to the Hermans (Equation (2)) and Segal methods (Equation (3)) showed for wood species that the crystallinity of the DIP and ITA species was higher than that of EUG and PIE, as presented in Table 4. These differences are confirmed when the values of the crystallite size along the (200) crystallographic plane are taken into consideration. Crystallinity indices increased with increasing crystallite sizes, because the crystallites surface corresponding to amorphous cellulose regions diminished [30]. These results indicated that DIP and ITA contain a more ordered cellulose structure than EUG and PIE. Curaua and ramie fibers present a higher crystallinity index when the Hermans method was used followed by jute and kenaf. Sisal and buriti present lower values. The crystallite sizes for curaua and ramie are also higher, which confirms that crystallinity indices increased with increasing crystallite sizes, which is associated with a reduction of the amorphous domains. The crystallinity index calculated using the Segal method showed similar values for jute, kenaf and ramie fibers, while demonstrating a surprising value for buriti. Notably, the value of the crystallinity index obtained by this method does not truly represent the percentage of crystalline parts in whole cellulose mass [31], because it only considers the crystallinity part present in the (200) plane, without considering the other two important crystallographic planes in (1–10) and (110). According to Xu and coworkers [31], the higher results from Segal’s method are not able to reflect the degree of biomass crystallinity, but instead provide a parameter for comparison. Figure 3 shows the liner regression applied for all fibers studied comparing the Hermans (Figure 3a) and Segal method, (Figure 3b). The linear regression shows a much better fit using Hermans’s method than the Segal method. The linear regression coefficient (R^2^) obtained for the crystallinity values from the Segal method was 0.04, while for Hermans’s method, the R^2^ value was 0.38. This result suggests that the Segal method is not a suitable method for determining the crystallinity index of lignocellulosic fibers and must be used with care.

**Table 4:** Parameters obtained from the XRD analysis of the fibers studied.

Fibers	*L* (200) (nm)	*Cr.I.* ^a^ (%)	*C.I.* ^b^ (%)	*Z*-values
*Eucalyptus grandis*	2.11	34.4	49.3	−25.7
*Pinus elliottii*	1.92	34.1	43.4	−32.9
*Dipteryx odorata*	2.18	43.0	55.7	−31.6
*Mezilaurus itauba*	2.23	37.8	52.7	−46.3
Curaua	3.43	60.6	43.5	−40.8
Jute	2.94	52.0	34.3	−18.0
Kenaf	2.71	51.1	34.9	−21.4
Ramie	3.31	56.5	34.8	−9.4
Sisal	3.37	47.1	57.3	−34.7
Buriti	3.70	45.1	71.2	−34.4

^a^ *Cr.I.*: crystalline index proposed by Hermans; ^b^ *C.I.:* crystalline index proposed by Segal.

There are two naturally occurring types of cellulose: cellulose I_α_ and I_β_. Only the I_β_ crystalline plane was considered in this analysis, because it is found in higher plants, whereas I_α_ is rather rare and found in bacterial and alga celluloses [30,32]. Cellulose I_β_ consists of parallel chains forming hydrogen-bonded sheets that stack with an alternating shear parallel to the axis of the chain stabilized by van der Waals interactions [30]. The I_α_ and I_β_ structures are assigned to triclinic and monoclinic unit cells, respectively [8,32]. As can be seen in Table 4, the *Z* values for wood and natural fibers indicate that the cellulose in these fibers was of the I_β_ dominant type.

### 3.4. Thermogravimetric Results

**Figure 4:**
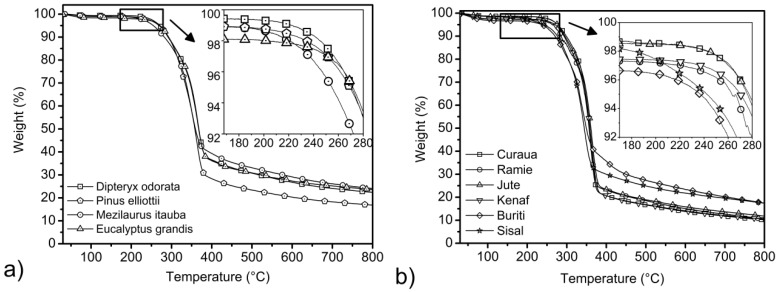
Thermogravimetric curves for the wood (**a**); and vegetal fibers (**b**) studied.

The thermal stability of wood flour and others vegetal fibers used as a filler or reinforcement in polymer composites is of paramount importance [33,34]. The manufacturing of composites requires the mixing of lignocellulosic materials and the polymer matrix at temperatures of around 200 °C for the most common thermoplastic polymers [33,35]. The degradation of lignocellulosic fibers due to high temperatures at the time of processing may lead to undesirable composite properties, such as odor and browning, along with a reduction in mechanical properties [33,35]. Hence, it is imperative that the degradation profile of the fibers must be determined prior to their use in composite applications [35]. The thermogravimetric curves for the wood species and natural fibers studied are presented in Figure 4.

Water loss is observed at around 100 °C for all fibers analyzed, and further thermal degradation takes place as a three-step process. In the first step, the degradation of hemicellulose takes place at around 300 °C. After that, a second weight loss occurs at around 350 °C, due to the main degradation of cellulose. Finally, the slow lignin degradation takes place between 250 and 600 °C. According to Kim *et al*. [36] the depolymerization of hemicellulose occurs between 180 and 350 °C, the random cleavage of the glycosidic linkage of cellulose between 275 and 350 °C and the degradation of lignin between 250 and 500 °C. The higher activity of hemicellulose in thermal decomposition might be attributed to its chemical structure [37,38]. Hemicellulose has a random amorphous structure, and it is easily hydrolyzed [37,38]. In contrast, the cellulose molecule is a very long polymer of glucose units, and its crystalline regions improve the thermal stability of lignocellulosic fibers [38]. Lignin is different from hemicellulose and cellulose, because it is composed of three kinds of benzene-propane units, being heavily cross-linked and having very high molecular weight [37,38]. The thermal stability of lignin is thus very high, and it is difficult to decompose [38].

As can be seen in detail in Figure 4a, at temperatures around 180–190 °C, the ITA wood showed a more significant weight loss. This behavior might be associated with the highest content of extractives in this wood, around 14%, as presented in Table 1. Extractives are compounds of lower molecular mass as compared to cellulose and can promote the ignitability of the wood at lower temperatures as a result of their higher volatility, and thus, they accelerate the degradation process. In this way, the degradation of one component may accelerate the degradation of the other wood components. As regards thermal degradation, the three major components of lignocellulosic fibers have their own characteristic properties based on the composition of cellulose, lignin and hemicellulose present in the different fibers. Therefore, the individual chemical components of the fibers behave differently if they are isolated or intimately combined within each single cell of the fiber structure. However, the DIP wood that contains around 11% of extractives showed higher thermal stability than ITA. This behavior may be related to the higher content of lignin in DIP relative to that of ITA and the higher crystallinity index and crystallite size in this wood, as can be seen in Table 4. It is also observed that buriti and sisal start to degrade at relatively lower temperatures. This might be associated with the bound water and respective extractive contents in these fibers, mainly for buriti. According to Table 1, buriti presents higher quantities of extractives when compared with the others natural fibers, corroborated by the two intense bands at 2918 and 2849 cm^−1^, which can be seen in Figure 1b and are probably related to organic extractives, as fatty acid methyl esters and phenolic acid methyl esters, that start the degradation process at relative low temperatures. Curaua and jute fibers present higher thermal stability than the other natural fibers, as can be seen in detail in Figure 4b and Table 5. The higher crystallinity index obtained for these fibers associated with lower quantities of bound water and extractives may be responsible for this.

**Table 5:** Thermal degradation temperature and residue at 800 °C for the fibers studied.

Fibers	*T*_i_ (°C) 3 wt% loss	*T* shoulder (°C)	DTG peak (°C)	Residue at 800 °C(%)
*Eucalyptus grandis*	250	291	364	23.6
*Pinus elliottii*	251	322	367	16.8
*Dipteryx odorata*	257	289	368	22.4
*Mezilaurus itauba*	237	275	350	24.1
Curaua	260	283	344	10.1
Jute	262	297	365	11.8
Kenaf	238	298	364	10.5
Ramie	221	289	357	10.6
Sisal	209	295	347	17.4
Buriti	92	291	334	17.5

The initial weight loss temperature, *T*_i_, of all fibers studied is considered as the temperature at which the sample loses 3% of its weight, as shown in Table 5. For wood, the lowest *T*_i_ value was attributed to ITA, as can be seen in detail in Figure 4a. This might be associated with the higher volatility of extractives and hemicellulose in this wood. The highest *T*_i_ values were observed for the EUG, PIE and DIP wood species. On the other hand, EUG, ITA and DIP had a significant amount of residue at 800 °C, probably due to the higher inorganic contents in these three wood species.

However, it is interesting to note that the thermal stability was more pronounced and the main decomposition of cellulose was higher for DIP compared to EUG and PIE. This behavior may be associated with the highest crystalline index and higher crystallite size of cellulose in this wood. In addition, the higher energy of hydrogen bond values in DIP in comparison with ITA (see Table 2) may indicate a more closed packaging cellulose structure that makes the heat transfer difficult, because the cellulose crystallinity domains act as barriers for the heat transfer, which possibly increases the wood thermal stability for this species. In a recent study, Kim *et al*. [30] showed that the thermal decomposition of cellulose shifted to higher temperatures with increasing crystallinity index and crystallite size.

Sisal and buriti presented the lowest T_i_ values, while curaua and jute showed the highest values in comparison to the other fibers. As discussed above, bound water and extractives can initiate the degradation process at relatively low temperatures and accelerate the degradation processes of the other fiber components, reducing the thermal stability of the fibers. On the other hand, higher cellulose crystallinity associated with higher crystallite size may act as barriers for the thermal degradation and increase the fiber thermal stability, as can be seen in Table 5, for curaua and jute fibers.

Therefore, a higher amount of hydrogen bonds between neighboring cellulose chains may be the result of a more packed cellulose structure, which can lead to higher crystallinity and thermal stability, as observed in the case of *Dipteryx odorata* and curaua fibers. In addition, intramolecular hydrogen bonds stabilize the cellulose molecules and may inhibit thermal expansion along the cellulose chains [39], improving the lignocellulosic fiber thermal stability. However, the decomposition of lignocellulosic fibers is a complex process and involves a series of competitive and/or consecutive reaction [33]. It might be difficult to distinguish and model the thermal decomposition behavior of each specific component in lignocellulosic fiber, due to the complexity of the growth of the fibers, which causes variance in component contents, crystal structure and chemical composition from one species to another [40,41,42].

## 4. Conclusions

The three methods used to characterize the fibers studied were found to be appropriate to evaluate differences in the structures of the fiber components. In the FTIR spectroscopy analysis, it was observed that the higher extractive contents in *Mezilaurus itauba* and buriti fiber might be associated with the prominent bands at around 2920 and 2850 cm^−1^, indicating that care needs to be taken when using these bands and other bands to compare crystallinity between lignocellulosic fibers using FTIR spectroscopy. The X-ray diffractometry results showed that *Dipteryx odorata* and curaua fiber contain much more cellulose chains in a highly organized form with a higher crystal size, which can lead to higher crystallinity. The combined results showed that lower quantities of extractives and bound water associated with higher crystallinity and higher crystallite size slow the degradation process and increase the thermal stability of lignocellulosic fibers.

## References

[1] Poletto M., Zeni M., Zattera A.J. (2012). Effects of wood flour addition and coupling agent content on mechanical properties of recycled polystyrene/wood flour composites. J. Thermoplast. Compos. Mater..

[2] Ornaghi H.L., Bolner A.S., Fiorio R., Zattera A.J., Amico S.C. (2010). Mechanical and dynamic mechanical analysis of hybrid composites molded by resin transfer molding. J. Appl. Polym. Sci..

[3] Romanzini D., Ornaghi H.L., Amico S.C., Zattera A.J. (2012). Influence of fiber hybridization on the dynamic mechanical properties of glass/ramie fiber-reinforced polyester composites. J. Reinf. Plast. Compos..

[4] Han G., Huan S., Han J., Zhang Z., Wu Q. (2014). Effect of acid hydrolysis conditions on the properties of cellulose nanoparticle-reinforced polymethylmethacrylate compostes. Materials.

[5] Klemm D., Heublein B., Fink H.-P., Bohn A. (2005). Cellulose: Fascinating biopolymer and sustainable raw material. Angew. Chem. Int. Ed..

[6] kerholm M., Hinterstoisser B., Salmén L. (2004). Characterization of the crystalline structure of cellulose using static and dynamic FT-IR spectroscopy. Carbohydr. Res..

[7] Oh S.Y., Yoo D.I., Shin Y., Kim H.C., Kim H.Y., Chung Y.S., Park W.H., Youk J.H. (2005). Crystalline structure analysis of cellulose treated with sodium hydroxide and carbon dioxide by means of X-ray diffraction and FTIR spectroscopy. Carbohydr. Res..

[8] Wada M., Okano T. (2001). Localization of I_α_ and I_β_ phases in algal cellulose revealed by acid treatments. Cellulose.

[9] Popescu M.-C., Popescu C.-M., Lisa G., Sakata Y. (2011). Evaluation of morphological and chemical aspects of different wood species by spectroscopy and thermal methods. J. Mol. Struct..

[10] Gümüskaya E., Usta M., Kirei H. (2003). The effects of various pulping conditions on crystalline structure of cellulose in cotton linters. Polym. Degrad. Stab..

[11] Faruk O., Bledzki A.K., Fink H., Sain M. (2012). Biocomposites reinforced with natural fibers: 2000–2010. Prog. Polym. Sci..

[12] Bledzki A.K., Gassan J. (1999). Composites reinforced with cellulose based fibres. Prog. Polym. Sci..

[13] Cordeiro N.C., Gouveia A.G.O., Moraes A.M., Amico S.C. (2011). Natural fibers characterization by inverse gas chromatography. Carbohyd. Polym..

[14] Satyanarayana K.G., Guimarães J.L., Wypych F. (2007). Studies on lignocellulosic fibers of Brazil. Part I: Source, production, morphology, properties and applications. Compos. Part A.

[15] Ornaghi H.L., Poletto M.P., Zattera A.J., Amico S.C. (2014). Correlation of the thermal stability and the decomposition kinetics of six different vegetal fibers. Cellulose.

[16] Poletto M.P., Zattera A.J., Santana R.M.C. (2012). Structural differences between wood species: Evidence from chemical composition, FTIR spectroscopy, and thermogravimetric analysis. J. Appl. Polym. Sci..

[17] Popescu C.-M., Singurel G., Popescu M.-C., Vasile C., Argyropoulos D.S., Willför S. (2009). Vibrational spectroscopoy and X-ray diffraction methods to establish the differences between hardwood and softwood. Carbohydr. Polym..

[18] Yokoi H., Nakase T., Goto K., Ishida Y., Ohtani H., Tsuge S., Sonoda T., Ona T. (2003). Rapid characterization of wood extractives in wood by thermal desorption-gas chromatography in the presence of tetramethylammonium acetate. J. Anal. Appl. Pyrolysis.

[19] Ishida Y., Goto K., Yokoi H., Tsuge S., Ohtani H., Sonoda T., Ona T. (2007). Direct analysis of phenolic extractives in wood by thermochemolysis-gas chromatography in the presence of tetrabutylammonium hydroxide. J. Anal. Appl. Pyrolysis.

[20] Mészáros E., Jakab E., Várhegyi G. (2007). TG/MS, Py-GC/MS and THM-GC/MS study of the composition and thermal behavior of extractive components of *Robinia pseudoacacia*. J. Anal. Appl. Pyrolysis.

[21] Popescu C.-M., Popescu M.-C., Vasile C. (2010). Structural changes in biodegraded lime wood. Carbohydr. Polym..

[22] Kondo T. (1997). The assignment of IR absorption bands due to free hydroxyl groups in cellulose. Cellulose.

[23] Struszczyk H. (1986). Modification of lignins III. Reaction of lignosulfonates with chlorophosphazenes. J. Macromol. Sci..

[24] Pimentel G.C., Sederholm C.H. (1956). Correlation of infrared stretching frequencies and hydrogen bon distances in crystals. J. Chem. Phys..

[25] Popescu C.-M., Popescu M.-C., Singurel G., Vasile C., Argyropoulos D.S., Willför S. (2007). Spectral characterization of eucalyptus wood. Appl. Spectrosc..

[26] Nelson M.L., O’Connor R.T. (1964). Relation of certain infrared bands to cellulose crystallinity and crystal lattice type. Part I. Spectra of types I, II, III and of amorphous cellulose. J. Appl. Polym. Sci..

[27] Oh S.Y., Yoo D.I., Shin Y., Seo G. (2005). FTIR analysis of cellulose treated with sodium hydroxide and carbon dioxide. Carbohydr. Res..

[28] Carrilo F., Colom X., Suñol J.J., Saurina J. (2004). Strucutral FTIR analysis and the thermal characterization of lyocell and viscose-type fibers. Eur. Polym. J..

[29] Corgié S.C., Smith H.M., Walker L.P. (2011). Enzymatic transformations of cellulose assessed by quantitative high-throughput fourier transform infrared spectroscopy (QHT-FTIR). Biotechnol. Bioeng..

[30] Kim U.-J., Eom S.H., Wada M. (2010). Thermal decomposition of native cellulose: Influence on crystallite size. Polym. Degrad. Stab..

[31] Xu F., Shi Y.-C., Wang D. (2013). X-ray scattering studies of lignocellulosic biomass: A review. Carbohydr. Polym..

[32] Poletto M., Pistor V., Zeni M., Zattera A.J. (2011). Crystalline properties and decomposition kinetics of cellulose fibers in wood pulp obtained by two pulping process. Polym. Degrad. Stab..

[33] Poletto M., Zattera A.J., Forte M.M.C., Santana R.M.C. (2012). Thermal decomposition of wood: Influence of wood components and cellulose crystallite size. Bioresour. Technol..

[34] Poletto M., Zattera A.J., Santana R.M.C. (2012). Thermal decomposition of wood: Kinetics and degradation mechanisms. Bioresour. Technol..

[35] Tserki V., Matzinos P., Kokkou S., Panayiotou C. (2005). Novel biodegradable composites based on treated lignocellulosic waste flour as filler. Part I. Surface chemical modification and characterization of waste flour. Compos. Part A.

[36] Kim H.-S., Kim S., Kim H.-J., Yang H.-S. (2006). Thermal properties of bio-flour-filled polyolefin composites with different compatibilizing agent type and content. Thermochim. Acta.

[37] John M.J., Thomas S. (2008). Biofibres and biocomposites. Carbohydr. Polym..

[38] Yang H., Yan R., Chen H., Zheng C., Lee D.H., Liang D.T. (2006). In-Depth investigation of biomass pyrolysis based on three major components: Hemicellulose, cellulose and lignin. Energy Fuels.

[39] Hidaka H., Kim U.-J., Wada M. (2010). Synchrotron X-ray fiber diffraction study on the termal expansion behavior of cellulose crystals in tension wood of Japanese poplar in the low-temperature region. Holzforsghung.

[40] Poletto M., Dettenborn J., Pistor V., Zeni M., Zattera A.J. (2010). Materials produced from plant biomass. Part I: Evaluation of thermal stability and pyrolysis of wood. Mater. Res..

[41] Yao F., Wu Q., Lei Y., Guo W., Xu Y. (2008). Thermal decomposition kinetics of natural fibers: Activation energy with dynamic thermogravimetric analysis. Polym. Degrad. Stab..

[42] Di Blasi C. (2008). Modeling chemical and physical process of wood and biomass pyrolysis. Prog. Energy Combust. Sci..

